# Dkk3/REIC Deficiency Impairs Spermiation, Sperm Fibrous Sheath Integrity and the Sperm Motility of Mice

**DOI:** 10.3390/genes13020285

**Published:** 2022-01-31

**Authors:** Ruizhi Xue, Wenfeng Lin, Hirofumi Fujita, Jingkai Sun, Rie Kinoshita, Kazuhiko Ochiai, Junichiro Futami, Masami Watanabe, Hideyo Ohuchi, Masakiyo Sakaguchi, Zhengyan Tang, Peng Huang, Yasutomo Nasu, Hiromi Kumon

**Affiliations:** 1 Department of Urology, Okayama University Graduate School of Medicine, Dentistry and Pharmaceutical Sciences, Okayama 700-8558, Japan; docfrankhsueh@gmail.com (R.X.); pbj35b7d@s.okayama-u.ac.jp (W.L.); jksun@surgery.cuhk.edu.hk (J.S.); masami5@md.okayama-u.ac.jp (M.W.); ynasu@md.okayama-u.ac.jp (Y.N.); 2 Department of Urology, Xiangya Hospital, Central South University, Changsha 410008, China; 3 Department of Cytology and Histology, Okayama University Graduate School of Medicine, Dentistry and Pharmaceutical Sciences, Okayama 700-8558, Japan; fujita00@md.okayama-u.ac.jp (H.F.); hohuchi@okayama-u.ac.jp (H.O.); 4 Department of Urology, Zhujiang Hospital, Southern Medical University, Guangzhou 510280, China; 5 Department of Cell Biology, Okayama University Graduate School of Medicine, Dentistry and Pharmaceutical Sciences, Okayama 700-8558, Japan; rie-k@okayama-u.ac.jp (R.K.); masa-s@md.okayama-u.ac.jp (M.S.); 6 Laboratory of Veterinary Hygiene, Nippon Veterinary and Life Science University, Tokyo 180-8602, Japan; kochiai@nvlu.ac.jp; 7 Department of Interdisciplinary Science and Engineering in Health Systems, Okayama University, Okayama 700-8530, Japan; futamij@cc.okayama-u.ac.jp; 8 Okayama Medical Innovation Center, Okayama University, Okayama 700-8558, Japan; 9 Innovation Center Okayama for Nanobio-Targeted Therapy, Okayama University, Okayama 700-8558, Japan; kumonhiro@gmail.com

**Keywords:** *Dkk3/REIC*, fibrous sheath, knock-out, RNA-seq, spermiation, sperm motility

## Abstract

The role of *Dickkopf-3 (Dkk3)/REIC* (*The Reduced Expression in Immortalized Cells*), a Wnt-signaling inhibitor, in male reproductive physiology remains unknown thus far. To explore the functional details of *Dkk3/REIC* in the male reproductive process, we studied the *Dkk3/REIC* knock-out (KO) mouse model. By examining testicular sections and investigating the sperm characteristics (count, vitality and motility) and ultrastructure, we compared the reproductive features between *Dkk3/REIC*-KO and wild-type (WT) male mice. To further explore the underlying molecular mechanism, we performed RNA sequencing (RNA-seq) analysis of testicular tissues. Our results showed that spermiation failure existed in seminiferous tubules of *Dkk3/REIC*-KO mice, and sperm from *Dkk3/REIC*-KO mice exhibited inferior motility (44.09 ± 8.12% vs. 23.26 ± 10.02%, *p* < 0.01). The Ultrastructure examination revealed defects in the sperm fibrous sheath of KO mice. Although the average count of *Dkk3/REIC*-KO epididymal sperm was less than that of the wild-types (9.30 ± 0.69 vs. 8.27 ± 0.87, ×10^6^), neither the gap (*p* > 0.05) nor the difference in the sperm vitality rate (72.83 ± 1.55% vs. 72.50 ± 0.71%, *p* > 0.05) were statistically significant. The RNA-seq and GO (Gene Oncology) enrichment results indicated that the differential genes were significantly enriched in the GO terms of cytoskeleton function, cAMP signaling and calcium ion binding. Collectively, our research demonstrates that *Dkk3/REIC* is involved in the process of spermiation, fibrous sheath integrity maintenance and sperm motility of mice.

## 1. Introduction

Infertility is a serious problem that affects over 14% of couples in the world, and half of the cases can be attributed to male factors [1]. With the help of modern techniques in molecular biology, genetic disorders have been proven to be a major cause of male infertility, especially for those with non-obstruction in the genital ducts [2]. To date, over 2000 genes have been reported to participate in male reproductive physiology, and new findings continue emerging [3]. According to recent studies, Wnt signaling, a classical intercellular pathway that regulates cellular proliferation and tissue development, plays a pivotal role in male reproductive function [4,5].

Among the Wnt signal community, *Dickkopf* (*Dkk*) family, a negative regulator of Wnt signaling, has recently attracted attention from scholars in male reproduction fields. Currently, five members of the family have been found, which are known as *Dkk1-4* and *Dkkl1* (*Dickkopf-like protein 1*) [6]. For years, our institution has been dedicated to the research of *Dkk3* and first identified its human homologous counterpart as a gene whose expression is reduced in immortalized cells, thus, also named *Dkk3* as *REIC* (*The Reduced Expression in Immortalized Cells*) [7]. 

*Dkk3/REIC* shares high sequence similarity with *Dkkl1,* and they are believed to arise from the same ancestral precursor [6]. There are several studies reporting that *Dkkl1* is deeply involved in the male reproductive process, as overexpressing *Dkkl1* in male mice can severely impair their spermatogenesis, and sperm from *Dkkl1*^−/−^ mice exhibit a decreased sperm-egg binding capacity [8,9].

To ascertain the functional characteristics of *Dkk3/REIC* in male reproductive physiology, we used *Dkk3/REIC* knock-out male mice as models and investigated the irregular reproductive findings. By conducting RNA sequencing (RNA-seq) of knock-out and wild-type mouse testes, we screened out the differentially expressed genes and preliminarily explored the potential molecular mechanism beneath the observed reproductive abnormalities.

## 2. Materials and Methods

### 2.1. Generation of Knock-Out Mouse Model and Genotype Identification

All animal procedures were approved by the Ethics Review Committee for Animal Experimentation of Okayama University (No. OKU2014608, OKU2016530). The welfare and treatment of the laboratory animals were based on the Guideline of Animal Experiment in Okayama University. The generation method of *Dkk3/REIC*-KO mice (general knock-out mouse) were described in our previous study [10]. Briefly, the mouse *Dkk3/REIC* gene from C57BL/6 genome was amplified. The exons 5 and 6 of *Dkk3/REIC* gene were flanked by *loxP* sites, which were subsequently deleted with Cre recombinase (Figure S1a). Then, the modified DNA clones were injected in Bruce-4 embryonic stem cells to form recombinant loci. 

Heterozygous male and female mice were bred to generate offspring whose genomic DNA was tested by multiplex touchdown PCR to verify the genotype using two forward primers (5′-AGACAGTGAGTGCTGTGGAGAC-3′ for WT and 5′-CAGTACTGGATCCGCTCCCAAT-3′ for KO) and one reverse primer (5′-AGGAGTGGAAATAAAGAGGACAGTG-3′ for WT and KO) (Figure S1b). The genomic DNA was purified with MagExtractor-Genome, and then PCR amplification was carried out using Quick Taq HS DyeMix (TOYOBO, Osaka, Japan) and the above primer set. Knock-out mice (*Dkk3/REIC*^−/−^) were maintained in a C57BL/6 background, and all animals were housed under a 12:12 light/dark cycle with sufficient food and water.

### 2.2. Periodic Acid Schiff (PAS) Staining

To prepare tissue sections, extracted testes were fixed with 10% formalin for 24 h, dehydrated with gradient ethanol and made transparent with dimethylbenzene. Then, the samples were embedded in paraffin, which were sliced with a thickness of 5 μm and reserved for later use. For PAS staining, the slides were first washed with conventional dewaxing procedures. After oxidation in 0.5–1% periodic acid aqueous solution (10 min), the slides were stained with Schiff’s reagent for 20 min. Next, sulphurous acid solution was added to achieve color differentiation, after which hematoxylin was used to stain the nuclei. Finally, the slides were dehydrated with ethanol and sealed with neutral balsam.

### 2.3. Immunofluorescence Staining

The testicular slides were dewaxed and then incubated in 10 mM sodium citrate buffer (pH 6.0) for 20 min in a 120 °C water bath to retrieve antigens. The slides were rinsed with TBS containing 0.1% Tween-20 and were blocked with goat serum for 60 min. Subsequently, the slides were incubated with anti-DKK3/REIC primary antibody (made by Okayama University) [11] overnight at 4 °C. The next day, the slides were rinsed with PBST and incubated with Alexa Fluor 546-conjugated secondary antibody (Thermo Fisher Scientific, Waltham, MA, USA) at room temperature for 40 min. After washing, the slides were treated with DAPI (Dojindo, Kumamoto, Japan) and then observed under LSM 780 confocal laser scanning microscope (Carl Zeiss Microscopy, Jena, Germany).

For the immunofluorescence staining of sperm, the epididymal sperm were extracted and made into sperm smears, which were subsequently formalin-fixed for 40 min at room temperature. Antigen blocking was performed using goat serum for 60 min. The smears were then incubated in anti-DKK3/REIC primary antibody overnight at 4 °C. Normal goat IgG was applied as a negative control (Figure S2). The use of secondary antibody and observation procedures were the same as with the testicular slides.

### 2.4. Assessment of Sperm Count, Vitality and Motility

Age-matched WT and KO mice (8–12 weeks) were sacrificed to compare sperm characteristics. Each side of dissected epididymis was incubated in 37 °C PBS (2 mL) for 10 min to let the epididymal sperm swim out. Then, 10 μL of the sperm solution was pipetted into a hemocytometer for the total count of sperm, and the average count of sperm per epididymis was calculated. Sperm vitality was assessed by Eosin staining and microscopic observation. Live spermatozoa were left unstained, and dead spermatozoa were stained pink or red. 

A total of 200 spermatozoa were evaluated, and the percentage of live spermatozoa was calculated. For sperm motility assessment, fresh sperm solution was diluted by 10 times (37 °C PBS), 10 μL of which was pipetted into a hemocytometer chamber, and then the sperm movement under a microscope was recorded as videos (10-s long). 

According to the assessing protocol of a previous study [12], the percentage of mobile sperm was estimated by observation of at least 200 sperm, and the motility of the sperm was defined as actively swimming or twitching. The counts (per seminiferous tubule) of specific testicular germ cells (including spermatogonial cells, spermatocytes, and spermatids) were assessed on PAS-stained slides. Each of the above assessments was conducted by the same skillful operator, and counts were repeated at least three times.

### 2.5. Transmission Electron Microscope (TEM) Examination

Sperm samples were fixed overnight by 2.5% glutaraldehyde. Subsequently, they were rinsed with PBS and postfixed with 1% osmium tetroxide for 30 min. Then, the sections were stained with uranyl acetate and lead citrate, followed by TEM (H-7650, HITACHI, Tokyo, Japan) examination.

### 2.6. Testicular RNA-Seq and GO Enrichment Analysis

The testes tissues of KO and WT mice (8 weeks) were isolated and fully ground separately. The total RNA was extracted using the RNeasy Plus Mini Kit (QIAGEN, Hilden, Germany). We used the poly-A selection protocol and Illumina TruSeq RNA library protocol to construct the RNA-seq libraries of KO and WT samples, respectively. Then, each library was sequenced on an Illumina HiSeq4000, platform (2 × 100 bp pair-end reads), following the manufacturer’s instructions. TopHat (v2.0.4) was applied to compare the read sequence with a mouse reference genome and ensemble transcriptome. 

The alignment results were then processed using edger (ver.3.16.5) for gene and transcript quantification. The trimmed mean of M-values (TMM) was calculated as the expression profile across differentially expressed genes (DEGs) and multiple test correction was conducted subsequently using the Benjamini–Hochberg method. DEGs were further screened by an FDR (false discovery rate) value < 0.05 and fold change ≥ 2. Gene Ontology (GO) term enrichment analysis was then performed using the DAVID (https://david.ncifcrf.gov/home.jsp, accessed on 20 June 2021). 

The raw sequencing reads data (fastq files) were deposited in the Sequence Read Archive (SRA) database (https://www.ncbi.nlm.nih.gov/sra/, accessed on 20 May 2021) with the accession numbers of SRR14606249 and SRR14606250.

### 2.7. Analysis of Public Single Cell RNA-Seq Data

Testicular single cell RNA-seq data of C57BL/6 mouse were downloaded from the Gene Expression Omnibus (GEO) database under the accession number GSE112393, and datasets of three male mice were used for analysis (7 to 9 weeks in age). Raw gene expression matrices were imported into RStudio (Version 1.4.1103) and converted into a Seurat object using the Seurat R package (Version 4.0.2). 

Cells with less than 500 expressing genes or more than 20% mitochondrial genome were discarded. After normalization and scaling, principal components (PCs) were calculated using the *RunPCA* function, and dimensionality reduction was achieved with the *RunUMAP* function. Finally, using the *FindCluster* function (resolution = 0.6), 11 clusters were found and further identified as different testicular cell types according to their expressed cell marker genes. The expression of *Dkk3/REIC* was demonstrated by *FeaturePlot*, *VlnPlot* and *DotPlot* functions.

### 2.8. Statistical Analysis

Statistical analyses by independent t test or Chi-square test as appropriate were performed using IBM SPSS Statistics 24.0 software. Each experiment was repeated at least three times, and the results are expressed as the mean ± SEM. Values of *p* < 0.05 were considered as statistically significant.

## 3. Results

### 3.1. Spermiation Failuare and Malposition of Differentiated Spermatids Exist in Testicular Sections of Dkk3/REIC-KO Mice

**Figure 1:**
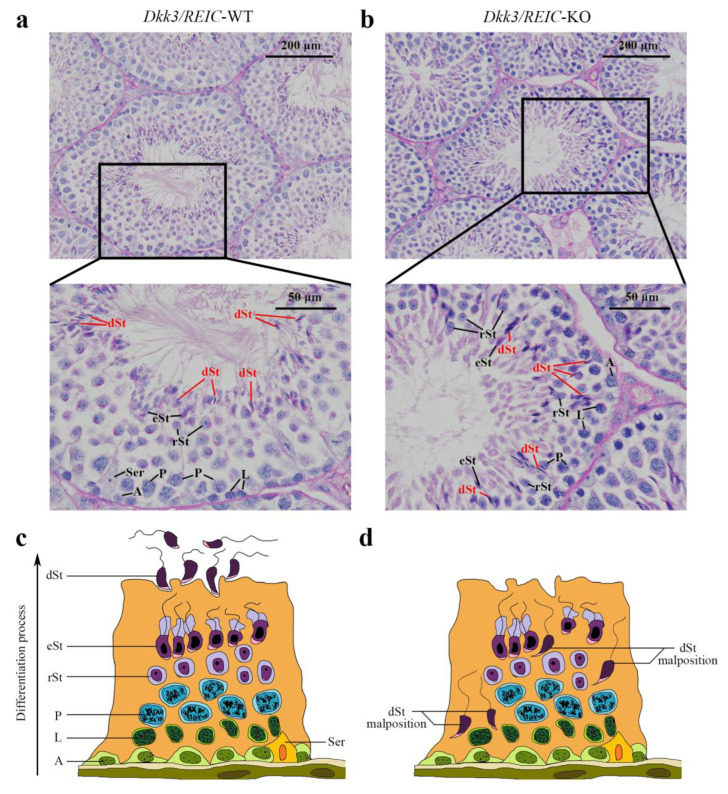
PAS staining of the testicular sections. (**a**) In the *Dkk3/REIC*-WT testicular section (stage VIII–IX of spermatogenesis), the distribution of germ cells was well arranged, and the tadpole-like differentiated sperm were uniformly located around the seminiferous tubule lumen waiting to be released. (**b**) In the *Dkk3/REIC*-KO testicular section (stage VIII–IX of spermatogenesis), however, the germ cell distribution was much disordered, few mature spermatids were in or surrounding the central lumen, while the differentiated sperm (marked by in red) irregularly wandered adjacent to the basement membrane. (**c**) The schematic drawing of typical spermatogenesis in the *Dkk3/REIC*-WT seminiferous epithelium. (**d**) The schematic drawing of pathological spermatogenesis in the *Dkk3/REIC*-KO seminiferous epithelium. Abbreviation: A = spermatogonia, Ser = sertoli cell, L = spermatocytes in leptotene phase, P = spermatocytes in pachytene phase, rSt = round spermatids, eSt = elongated spermatids and dSt = differentiated spermatids.

To investigate morphological changes of spermatogenesis after *Dkk3/REIC* was knocked out, the PAS-stained testicular sections of *Dkk3/REIC*-KO and *Dkk3/REIC*-WT mouse were examined. We noticed that spermiation failure existed in *Dkk3/REIC*-KO mice testes, and disordered placement of differentiated spermatids (which was most clearly displayed in stage VIII–IX) was commonly found in their testicular seminiferous tubles. As shown in Figure 1, differentiated spermatids were absent from the centers of the tubules and irregularly wandered adjacent to the basement membrane. However, the average amount of testiclular germ cells revealed no significant difference between the *Dkk3/REIC*-WT and *Dkk3/REIC*-KO mice (Table S1).

### 3.2. Expression and Distribution of Dkk3/REIC in Testes and Sperm of Male Mouse

**Figure 2:**
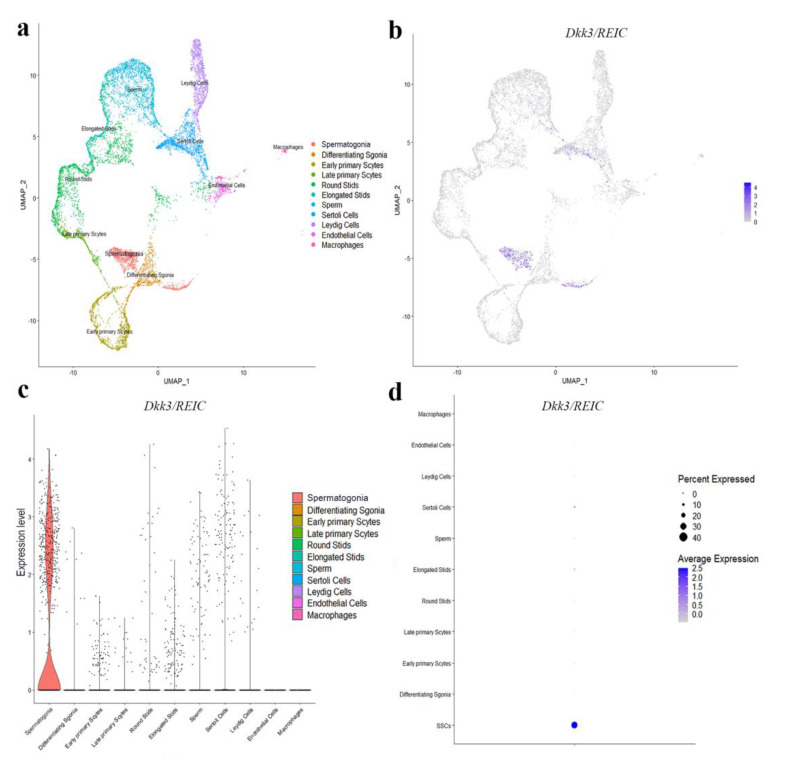
Distribution of *Dkk3/REIC* among testicular cell clusters in public scRNA-seq transcriptome atlas. (**a**) Analysis revealed 11 clusters that were matched to 11 testicular cell types. (**b**) The FeaturePlot function, (**c**) VlnPlot function and (**d**) DotPlot function of Seurat packages demonstrated the distribution of *Dkk3/REIC* among these different cell types. The results indicated that *Dkk3/REIC* had the highest level in spermatogonia.

To explore the transcript expression of *Dkk3/REIC* in mouse testes, we used the published C57BL/6 testicular scRNA-seq transcriptome in GEO database (GSE112393) and assessed the cell-specific expression of *Dkk3/REIC*. Clustering analysis identified 11 types of testicular cells (Figure 2a), *Dkk3/REIC* transcripts existed in most of the cell types except for macrophages and endothelial cells (Figure 2b,c), and this was predominately expressed in spermatogonia cells (Figure 2d). Then, we used testicular sections and sperm smear obtained from KO and WT mice and detected Dkk3/REIC expression by immunofluorescence (IF) staining. 

**Figure 3:**
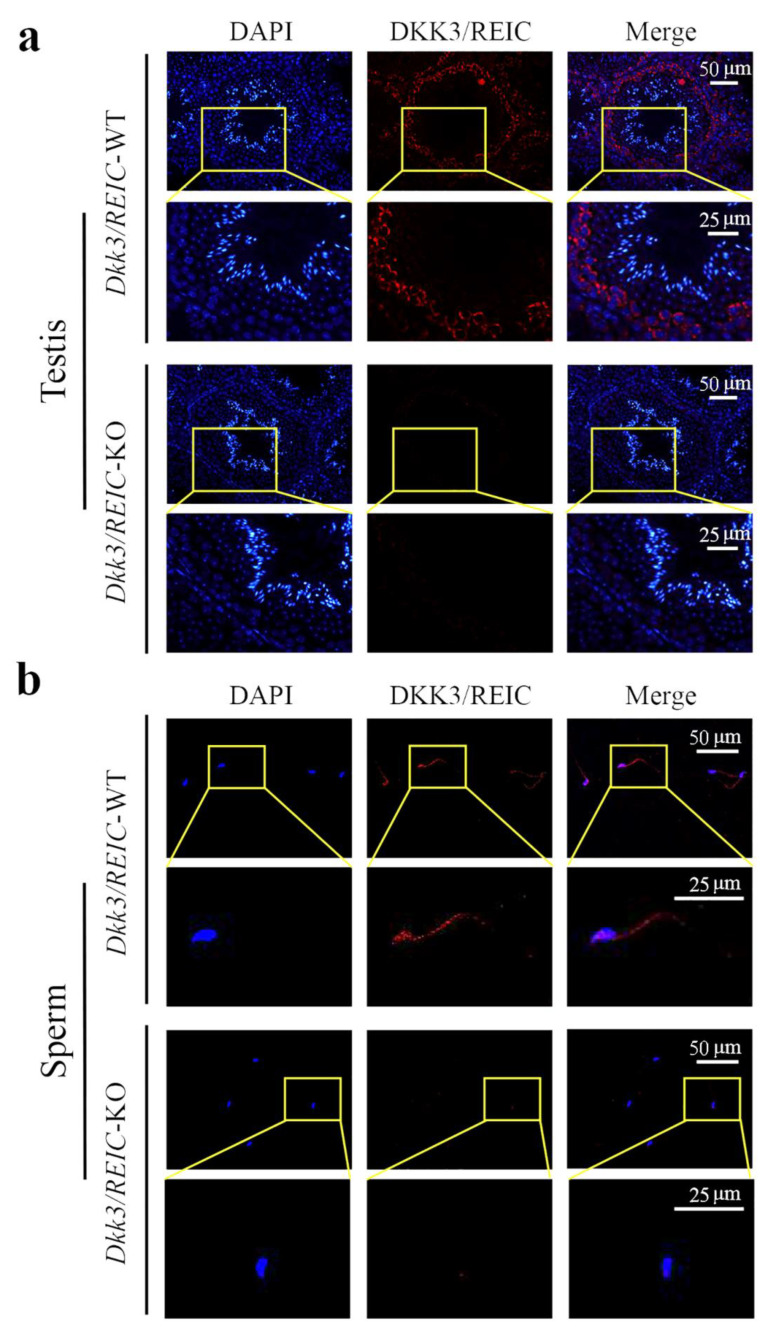
Expression and localization of DKK3/REIC protein in mouse seminiferous tubules and sperm. The expression and distribution of DKK3/REIC protein in (**a**) testes and (**b**) sperm of *Dkk3/REIC*-WT and *Dkk3/REIC*-KO mice.

As shown in Figure 3a, DKK3/REIC in the testicular section was mainly expressed in spermatogonia. This was compliant with our results from the published scRNA-seq data. Figure 3b shows the distribution of DKK3/REIC in mouse sperm, which was extensively expressed in both the head and tail, indicating that it may be a functional protein for spermatic activity.

### 3.3. Sperm Motility Is Impaired in Dkk3/REIC-KO Male Mice While the Sperm Total Count and Vitality Rate Show No Significant Difference

As documented in Table S2, although the average epididymal sperm count (per epididymis) of *Dkk3/REIC*-KO mice was nearly 1 million less than their wild-type counterparts (9.30 ± 0.69 vs. 8.27 ± 0.87, ×10^6^), the difference was not statistically significant (*p* > 0.05). Sperm vitality rates between the WT and KO mice were also non-significant (72.83 ± 1.55% vs. 72.50 ± 0.71%, *p* > 0.05). Irregular findings were noticed in the motility status of *Dkk3/REIC*-KO mice sperm. Compared with the wild-types, KO mice showed a significant lower proportion of motile sperm (44.09 ± 8.12% vs. 23.26 ± 10.02%, *p* < 0.01).

### 3.4. Ultrastructural Examination Reveals Defects in Flagellum Fibrous Sheath of Dkk3/REIC-KO Mouse Sperm

**Figure 4:**
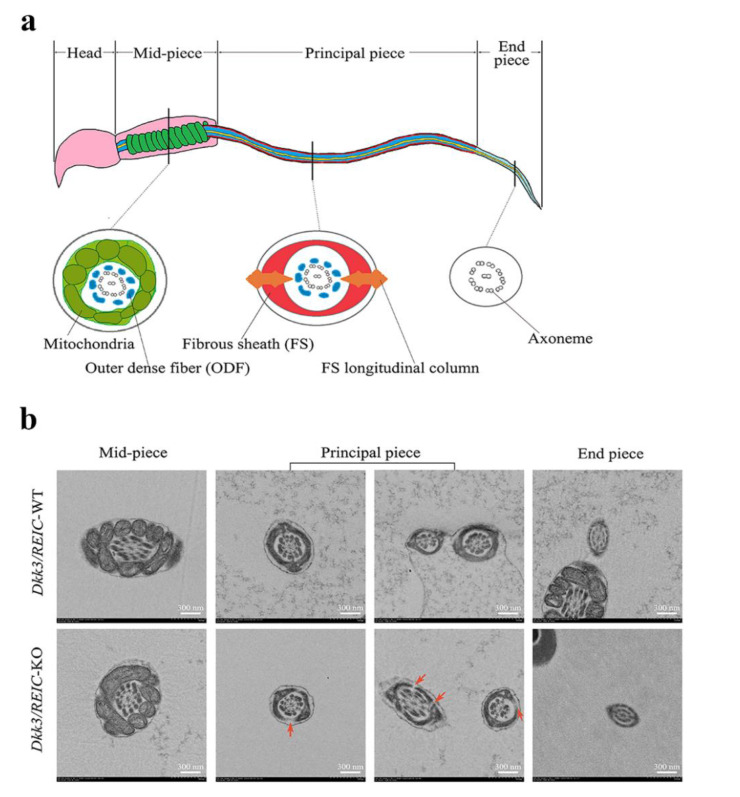
Transmission electron microscope observation of *Dkk3/REIC*-WT and *Dkk3/REIC*-KO sperm. (**a**) Schematic drawing of the mouse sperm ultrastructure. The sperm flagellum can be divided into three parts, which are the mid-piece, principal piece, and end piece. Their cross section views are different from each other, and the fibrous sheath structure mainly exists in the principal piece. (**b**) The TEM images of *Dkk3/REIC*-WT and *Dkk3/REIC*-KO sperm. No obvious difference was found between the mid-piece and end piece; hwoever, the principal piece of *Dkk3/REIC*-KO sperm showed irregular fractures in the fibrous sheath (arrows).

The normal sperm structure under TEM is depicted in Figure 4a. Sperm flagellum can be routinely divided into three sub-parts, which are the mid-piece, principal piece and end piece [13]. The cross sections of different sub-parts contain different structures. For *Dkk3/REIC*-KO sperm, irregular changes were found in the fibrous sheath, a major component of the principal piece. Compared with their wild-type counterparts, which showed an intact fibrous sheath, *Dkk3/REIC*-KO sperm had multiple defects and fractures in the fibrous sheath of the flagellum (Figure 4b). Such abnormalities could disrupt the structural continuity of the sperm flagellum, which might be linked with the sperm motility impairment in *Dkk3/REIC*-KO mice.

### 3.5. RNA Sequencing of Testicular Transcriptome from Dkk3/REIC-WT and Dkk3/REIC-KO Mice and GO Enrichment Analysis

**Figure 5:**
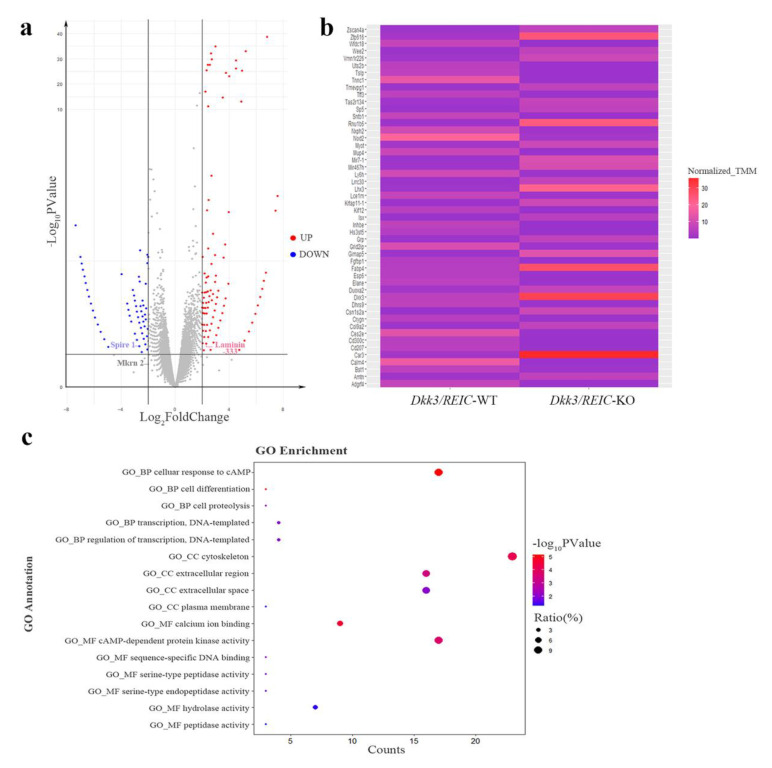
Differentially expressed genes and enrichment results of *Dkk3/REIC*-WT and *Dkk3/REIC*-KO mice. (**a**) Volcano plot of differentially expressed genes (DEGs). Genes with significant difference (FDR < 0.05 and fold change ≥ 2) are marked in colors. Red and blue, respectively, indicate the up-regulated and down-regulated genes in *Dkk3/REIC*-KO mice. DEGs of *Laminin-333*, *Spire 1* and *Mkrn 2* are marked by labels. (**b**) The relative expressions of the top 50 DEGs among *Dkk3/REIC*-WT and *Dkk3/REIC*-KO mice testes. (**c**) The top 200 DEGs analyzed by Gene Ontology (GO) enrichment.

The RNA-seq of testes transcriptome revealed 36,060 DEGs. The DEGs with FDR value less than 0.05 and a fold change larger than 2 are marked by colors in the volcano plot (Figure 5a). The relative expressions of top 50 DEGs between *Dkk3/REIC*-WT and *Dkk3/REIC*-KO testes are shown in a heatmap (Figure 5b). Then, we selected the top 200 DEGs as candidate genes and conducted GO (Gene Ontology) enrichment analysis. As shown in Figure 5c, for the GO category of Biological Process (BP), the candidate genes were predominately enriched in the *cellular response to cAMP*. 

In the Cellular Component (CC) category, candidates were significantly concentrated in the *cytoskeleton*. For the Molecular Function (MF) category, *calcium ion binding* and *cAMP-dependent protein kinase activity* were the most significantly enriched GO terms. Collectively, the GO enrichment results indicated that the functions of the selected DEGs were mainly focused on cAMP signaling, calcium ion binding and cytoskeleton function, revealing that the absence of *Dkk3/REIC* may induce changes in the above activities and consequently lead to reproductive impairment.

## 4. Discussion

The role of Wnt signaling in the male reproductive system has long concerned researchers. Most members of this signaling pathway have been proven to function in spermatogenesis and sperm maturation (the details are summarized in our previous review [4]). Nevertheless, there is still a knowledge gap in the reproductive function of certain Wnt signals, such as *Dkk3/REIC*. As a member of *Dickkopf* family, *Dkk3/REIC* shares high sequence similarity with *Dkkl1*, another *Dickkopf* family member, which has been reported to participate in acrosome reaction and sperm motility during fertilization [8,9]. 

The current findings regarding *Dkkl1* suggest that *Dkk3/REIC* may also play a role in male reproductive process, and our research aims to determine the functional details of *Dkk3/REIC* in sperm physiology. *Dkk3/REIC* was reported to be expressed in primate testicular Sertoli cells [14]. In mice, however, *Dkk3/REIC* is mainly present in the spermatogonia of seminiferous tubules according to a previous study [11], which is consistent with our scRNA-seq data results and immunofluorescence outcomes. 

The different findings between primate and mouse testes indicate that *Dkk3/REIC* expression may diversify among different species, and its distribution in the human reproductive system deserves investigation. Our research revealed spermiation failure and inappropriate placement of differentiated spermatids in seminiferous tubules of *Dkk3/REIC*-KO mice. 

Spermatogenesis is a continuous and multistage process, through which the peripheral spermatogonial cells gradually migrate to the interior and finally differentiate into spermatids and are released into the central lumen of the seminiferous tubules. Based on the changes in the morphology of acrosome and nucleus, the differentiating process in seminiferous tubules is routinely divided into 12 stages for mice. Stage VIII is commonly used for assessing spermiation, because, starting from this stage, differentiated spermatids are released into the lumen as spermatozoa [15].

Previous evidence proved that germ cell migration within seminiferous tubules is regulated by a specialized adhesion junction termed the ectoplasmic specialization (ES), and abnormal positioning of sperm within the epithelium has been frequently observed in mice with ES defects [16]. Microcosmic studies revealed that ES determines the orientation, translocation and spermiation of spermatids in the epithelium by controlling cytoskeleton features, such as microtubules and motor proteins [17]. 

In this study, RNA-seq and GO enrichment analysis indicated that knocking out *Dkk3/REIC* could implicate *cytoskeleton* malfunction, and DEGs, such as *Laminin-333*, *Spire 1* and *Mkrn 2* (Figure 5a), have been previously reported as upstream of ES genes [18,19,20]. Thus, *Dkk3/REIC* may interact with ES to regulate spermiation in seminiferous tubules. However, the results of the sperm total count appear to conflict with the observation of spermiation failure. 

Although the total sperm count of KO mice decreased in all three groups that we examined, the difference was not statistically significant. This may be related to the fact that the sperm-releasing function of pathologic tubules is not completely disabled, and there are still some atypical lesions with ongoing spermiation in *Dkk3/REIC*-KO mice, which may compensatively close the gap with the wild-types.

The fibrous sheath is a cytoskeletal structure that surrounds the sperm flagellum and maintains its stability during movement. We observed sperm fibrous sheath defects along with a decline of sperm motility in *Dkk3/REIC*-KO mice, and such ultrastructural pathology has been reported in previous studies of other knock-out mouse models [13,21]. RNA-seq analysis indicated that cAMP signaling and calcium ion binding were the significantly enriched GO terms in addition to the cytoskeleton. 

According to previous studies, cAMP-dependent protein kinase (cPKA) is among the components of the sperm fibrous sheath [22]. One of cPKA’s subunits (the RII subunit) can bind to A-kinase anchoring proteins (AKAPs)—the main functional protein of the sperm fibrous sheath—to form a tightly associated complex [23]. Therefore, some scholars deem cPKA to be a scaffolding element in the assembly of the fibrous sheath [22]. Combining such evidence with our findings of sperm ultrastructure, we believe there is a possibility that sperm fibrous sheath defects were caused by the AKAPs and cPKA abjunction due to the absence of DKK3/REIC.

In addition to the integrity of the fibrous sheath, sperm motility also depends directly on cAMP and calcium functioning. cAMP has been reported as an essential element in the initiation of sperm motility, as it can activate tyrosine kinase and increase the flagellar beat frequency [24]. Past studies have thoroughly investigated the role of calcium in sperm motility, indicating that increasing Ca^2+^ concentration can raise the sperm glycolysis level and promote motility hyperactivation [25]. Based on the above evidence, *Dkk3/REIC* may also influence sperm motility by regulating cAMP and calcium function.

There are some limitations in our research. First is the limited sample size. Owing to the shortage of available *Dkk3/REIC*-KO mice, the functional role of *Dkk3/REIC* in other reproductive aspects, such as sex hormone secretion, acrosome reaction and sperm-oocyte binding, remains to be explored. In addition, due to the technical difficulties of sperm protein extraction, proteomic analysis of *Dkk3/REIC*-KO sperm is still pending, and we hope to make up the above gaps in our future work. In conclusion, *Dkk3/REIC* is expressed in the mouse reproductive system, and it affects spermiation and is required for maintaining the normal motility and fibrous sheath integrity of sperm.

## Data Availability

Testicular single cell RNA-seq data of C57BL/6 mouse were downloaded from the Gene Expression Omnibus (GEO) database (https://www.ncbi.nlm.nih.gov/geo/, accessed on 10 June 2021) with the accession number GSE112393. The bulk sequencing data in this study have been deposited in the Sequence Read Archive (SRA) database (https://www.ncbi.nlm.nih.gov/sra/, accessed on 20 May 2021). The accession numbers are SRR14606249 and SRR14606250. Patient consent is not applicable for this study has not involved humans.

## Supplementary Materials

The following supporting information can be downloaded at: https://www.mdpi.com/article/10.3390/genes13020285/s1, Figure S1: Genotype verification of *Dkk3/REIC*-KO mice by PCR; Figure S2: Normal goat IgG applied as a negative control for anti-*DKK3/REIC* primary antibody in sperm immunofluorescence staining; Table S1: Comparison of the testicular germ cell amount (counts/per seminiferous tubule) between *Dkk3/REIC*-WT and *Dkk3/REIC*-KO male mice; Table S2: Sperm characteristics comparison between *Dkk3/REIC*-WT and *Dkk3/REIC*-KO male mice.
