# Supplementary material for: Dkk3/REIC Deficiency Impairs Spermiation, Sperm Fibrous Sheath Integrity and the Sperm Motility of Mice
Source: Genes (Basel). 2022 Jan 31;13(2):285. doi: 10.3390/genes13020285 (PMC8872165; doi:10.3390/genes13020285)
Supplement: Supplementary file 1 [file genes-13-00285-s001.zip › genes-1565229-supplementary.pdf]

Supplementary Material

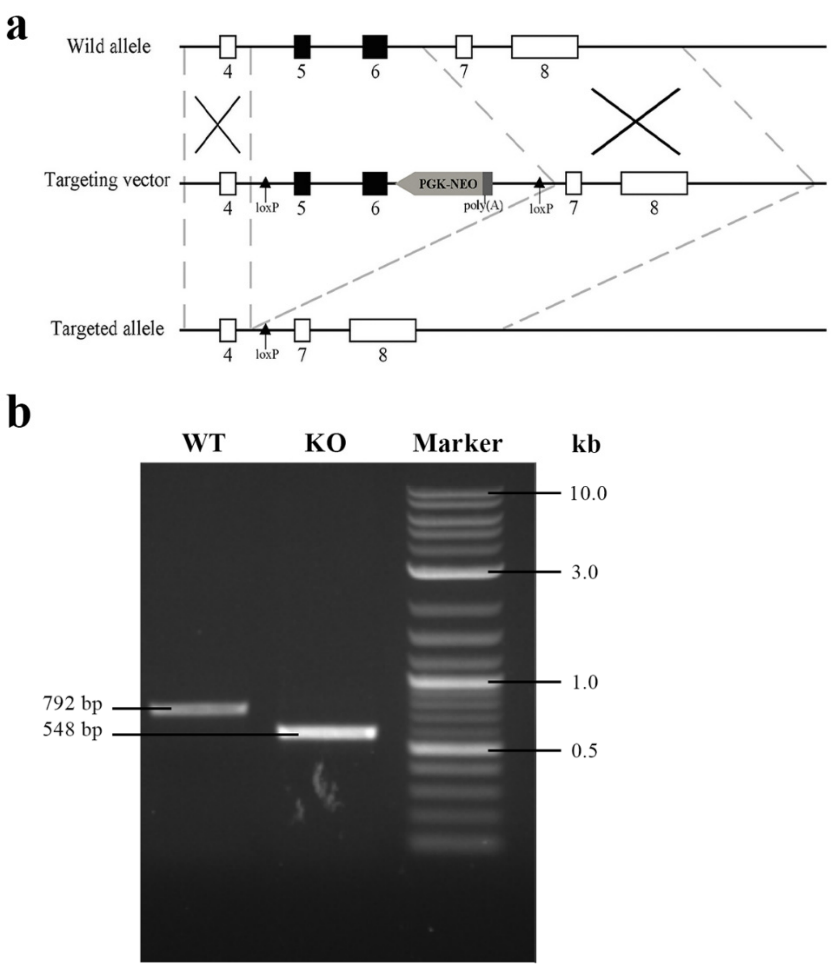

Figure S1. Genotype verification of *Dkk3/REIC*-KO mouse by PCR

(**a**) Gene editing strategy was sketched by the schematic diagrams of the wild type allele, targeting vector and targeted allele. The numbered boxes represent *Dkk3/REIC* exons. The exon 5 and exon 6 are wrapped by *loxP* sequence and cut by PGK/neomycin cassette. (**b**) The genomic DNA from offspring of *Dkk3/REIC* heterozygous mice was tested by multiplex touchdown PCR to verify their genotypes. The deletion of *Dkk3/REIC* alleles is confirmed when the band size showed as 548 bp.

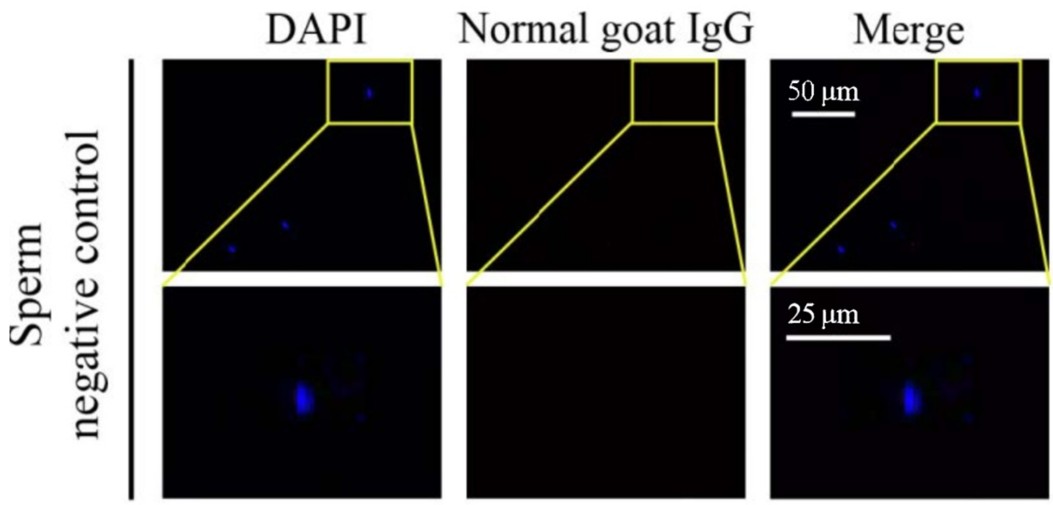

Figure S2. Normal goat IgG is applied as a negative control for anti-DKK3/REIC primary antibody in sperm immunofluorescence staining

Table S1 Comparison of testicular germ cell amount (counts / per seminiferous tubule) between *Dkk3/REIC*-WT and *Dkk3/REIC*-KO male mice

	n	Spermatogonial cells	Spermatocytes	Spermatids
Dkk3/REIC-WT	3	94.00 ± 7.21	171.33 ± 22.74	231.67 ± 19.09
Dkk3/REIC-KO	3	85.33 ± 15.01	147.33 ± 23.86	205.00 ± 19.00
p value		> 0.05	> 0.05	> 0.05

n = number of mice

Table S2 Sperm characters comparison between *Dkk3/REIC*-WT and *Dkk3/REIC*-KO male mice

	n	Sperm count ($\times 10^6$)	Vitality (%)	Motility (%)
Dkk3/REIC-WT	3	9.30 ± 0.69	72.83 ± 1.55	44.09 ± 8.12
Dkk3/REIC-KO	3	8.27 ± 0.87	72.50 ± 0.71	23.26 ± 10.02
p value		> 0.05	> 0.05	< 0.01

n = number of mice
